# Chandipura Virus Induced Neuronal Apoptosis via Calcium Signaling Mediated Oxidative Stress

**DOI:** 10.3389/fmicb.2018.01489

**Published:** 2018-07-06

**Authors:** Abhishek K. Verma, Sourish Ghosh, Anirban Basu

**Affiliations:** National Brain Research Centre, Manesar, India

**Keywords:** CHPV- Chandipura virus, MAPK- mitogen-activated protein kinase, ROS- reactive oxygen species, apoptosis, calcium signaling

## Abstract

Chandipura Virus (CHPV) a negative-stranded RNA virus belonging to the *Rhabdoviridae* family, has been previously reported to bring about neuronal apoptosis by stimulating oxidative stress. Our *in silico* data suggested the involvement of Angiotensin II in intracellular Ca^2+^ secretion within CHPV infected cells that further lead to enhancement of ROS level and mitochondrial dysfunction. ROS is also known to phosphorylate p38 that leads to neuronal apoptosis through FasL-FADD pathway during CHPV infection. Minocycline a broad-spectrum antibiotic well-known for its anti-oxidative and anti-inflammatory role was used in the present study to investigate its efficacy against CHPV. The results obtained from the present study showed minocycline to be effective in mitigating the levels of cytoplasmic Ca^2+^, ROS, phosphorylation of p38 molecules and hence cellular apoptosis. Thus minocycline apart from being an anti-inflammatory and anti-oxidative agent, our study showed that minocycline has an additional Ca^2+^ chelation activity.

## Introduction

Apoptosis or programmed cell death is an important mechanism that plays a crucial role in host-virus interactions (Irurzun et al., 1995; Lin et al., 2016). Either the process is induced by the virus to complete their life cycle within their hosts, or the host induces cellular apoptosis to limit the viral spread. Over the years researchers have been trying to decipher this inter-relationship between the host, virus, and process of apoptosis, but till date, no definite conclusion has been drawn on this. Cellular apoptosis may be induced by external death domain receptor through Fas/CD95 recruiting FADD (Fas-associated protein with death domain) or intrinsically by cellular signaling involving mitochondria (Waring and Müllbacher, 1999; Elmore, 2007). It has also not been determined that which are the key players in deciding through which apoptotic pathway the host-virus interaction will operate.

Chandipura virus (CHPV) belonging to the Rhabdoviridae family is an arbovirus and has been a severe threat to the population in the Indian subcontinent for more than a decade (Menghani et al., 2012). It has been reported to induce Fas-mediated apoptosis through FasL-FADD pathway activation (Ghosh et al., 2013), but the mechanism for FasL generation post-CHPV infection is still elusive. Increase in intracellular Calcium (Ca^2+^) level has been reported in case of other viral infections that lead to ROS generation and formation of free radicals (Dayanithi et al., 1995; Zhou et al., 2009) although the molecular mechanism involving this surge in intracellular Ca^2+^ is not well understood. It has been reported from our lab previously that reactive oxygen species (ROS) induced oxidative stress is a critical factor for apoptosis following CHPV infection (Ghosh et al., 2016). Hence in order to understand the pathology of CHPV infection in brain involving neurodegeneration, it is essential to determine factors and conditions that regulate the ROS generation in response to an intracellular rise in Ca^2+^.

It has been reported that mitochondrial ROS generation leads to activation of Mitogen-activated protein kinases (MAPK) (Drigotas et al., 2013; Son et al., 2013) pathways that further results in phosphorylation of p38 signaling molecule that is an important player in apoptosis induction through FasL production (Dermitzaki et al., 2002). The activation of p38 MAPK has been observed in some physiological responses such as apoptosis of myocardial cells and adipogenesis in 3T3-L1 cells. Moreover, early membrane blebbing during oxidative stress-induced apoptosis is tightly regulated by p38 MAPK-mediated actin organization. Although p38 MAPK has been reported to be involved in various physiological events, the mechanism of activation of p38 MAPK by various stimuli as well as its biological roles remain to be elucidated especially in cases of viral infection.

Minocycline is a broad-spectrum tetracycline antibiotic that has been proven to be effective against viral infection (Szeto et al., 2010; Dutta and Basu, 2011). There have been reports describing its anti-viral (Dutta and Basu, 2011), antioxidant (Chen et al., 2012), etc. roles. In the present investigation, we studied the various molecular interactors of minocycline within the neurons post CHPV infection and reported how they are modulated by the drug to hinder the successful replication of the virus within their host.

## Materials and Methods

### Ethics Statement

All animal experiments were approved by the Institutional Animal and Ethics Committee of the National Brain Research Centre (approval no. NBRC/IAEC/2013/88). The animals were handled in strict accordance with good animal practice as defined by the Committee for Control and Supervision of Experiments on Animals, Ministry of Environment and Forestry (CPCSEA), Government of India.

### Virus and Cell

CHPV (strain no. 1653514 isolated from the human patient in Nagpur, 2003) was propagated in Vero cell line. The virus was propagated in the Vero cell line, and viral titer was measured using plaque assay, which was found to be 3 × 10^9^ pfu/ml. HT22 (immortalized mouse hippocampal neuronal cell line was gifted by Dr Shiv Kumar Sharma, National Brain Research Centre) cells were used for our experiment with prior permission from Dr Dave Schubert of Salk Institute from whom these cells were initially obtained. HT22 cells were grown at 37°C in Dulbecco’s modified Eagle medium (DMEM) supplemented with 3.7%, Sodium bicarbonate (Sigma, United States), 10% FBS (Gibco, Thermo Fisher Scientific, United States) and penicillin-streptomycin (Sigma, United States).

### MTT Assay

The viability of cultured cells was determined by-(4,5-dimethylthiazol-2-yl)-2,5-diphenyltetrazolium bromide (MTT; Sigma) as described earlier (Ghosh et al., 2009). HT-22 was seeded in triplicate at a density of 2 × 10^4^ cells per well on a 96-well plate. After 12 h, cells were treated with varying concentrations (25-150 μM) of minocycline in a serum free condition for another 18 h. MTT solution (0.5 mg/ml) was then added to each well and incubate for 4 h at 37°C. At the end of the incubation period, the medium was removed and the resulting purple formazan was solubilized with acidic isopropanol (0.1 N HCl in absolute isopropanol), and the absorbance was read at 570 nm using Biorad Microplate reader (Biorad, United States).

### Infection and Treatment of Cells

HT22, hippocampal neuronal cells were used for experimental purpose. HT22 cells were cultured in 10% serum containing media and were seeded in 60 mm plate at the density of 5 × 10^5^. After 12 h the media was replaced by serum-free media to limit the growth rate of the cells so that a particular number of cells can be monitored for infection. Post 2 h incubation HT22 cells were infected with CHPV at the multiplicity of infection (MOI) 0.1 for 1 h and then cells were washed twice with 1X PBS to remove non internalized virus present in media and then, minocycline was added at the concentration of 75 μM. Cells at various post-infection time points were harvested, and the culture supernatants were collected and stored at -80°C.

### Plaque Assay

Plaque assay was performed following the previously published protocol from our lab. Vero cells were cultured in 10% FBS containing DMEM and seeded in 6 well plates at the density of 4 × 10^4^ cells/well. After complete monolayer formation was achieved, serum containing media was changed to serum-free media and incubated for 2 h to acclimatize the cells for serum starvation. Meanwhile, the serial dilution was prepared in serum-free DMEM starting with a 1:10 dilution of the stock solution (by adding 100 μL of samples of each group in 900 μL media). The stock solution was serially diluted using 10-fold dilutions. Each dilution was added to each well of Vero cells. Post 2 h of incubation with the respective dilutions at 37°C; supernatants were removed and washed twice with 1X PBS to avoid multiple infection cycles. 3 ml of agarose overlay [9 ml 2% agarose (Roche, Germany), 10 ml 2X Minimal essential media (Sigma, United States), 1 ml FBS (Gibco, Thermo Fisher Scientific, United States), 100 μL penicillin-streptomycin (Sigma, United States)] was then added to each well. The plate was kept at 4°C for solidifying the overlay after which it was returned to 37°C for incubation of 24 h. 4% PFA was added post-incubation period for fixation of the cells for further analysis. Subsequently, the overlay was removed, and cells were stained with crystal violet and plaque was counted. The viral titers were expressed as PFU/ml, calculated as [(number of plaques per well) × (dilution)]/(inoculum volume).

### TUNEL Assay

HT22 cell lines were plated at a density of 10^5^ cells/well in 4-well chamber slide with serum containing media. Different samples were treated with 1X PBS, CHPV, and Minocycline. At 12 h post-infection period, cells were subjected to *In situ* Cell Death. Detection Kit, TMR red as per the manufacturers’ guidelines (Roche, Germany). Similarly, mouse brain sections were processed for TUNEL staining. Cell counting was done manually using ImageJ software.

### Immunoblotting

HT22 cells under different treatment conditions were harvested for obtaining total cellular extracts, and the protein isolation procedure and immunoblotting steps were performed according to standard procedure. After being blocked with 5% skimmed milk, the membranes were incubated with primary antibodies against cleaved caspase 3 (Abcam, United States), Cleaved Caspase 8 (Cell signaling, United States), FADD (Sigma, United States), p-PLC-γ (Cell signaling, United States), p-p38(Cell signaling, United States), CHPV (a kind gift by Bharat Biotech International Limited, Hyderabad, India) at 1:1,000 dilutions. After extensive washes with 0.1% PBS-Tween, blots were incubated with the Anti-Rabbit peroxidase-conjugated secondary antibodies (Vector Laboratories, United States). The blots were processed for development using chemiluminescence reagent (Millipore, United States). The images were captured and analyzed using the Uvitec Cambridge using NineAlliance software (Uvitec, United Kingdom). β-actin antibody (Sigma, United States) at 1:10,000 dilution was used as loading control.

### ROS Measurement

Intracellular ROS generation in Mock infected and treated cells was assessed using the cell permeable, non-polar H_2_O_2_ sensitive dye 5-(and-6)-chloromethyl-2′, 7′ - dichloro dihydro fluorescein diacetate (CM-H2DCFDA) (Sigma Aldrich, United States) as described previously (Verma et al., 2016). The extent to which H_2_O_2_ is generated is defined as the extent of ROS generation. Briefly, HT22 cells of different group, i.e. mock, CHPV infected and minocycline group were cultured, and then in serum-free media it was infected and treated with minocycline. This was further followed by incubation in serum-free media for 3 h after which, the cells were further treated with H2DCFDA (1 μM) for 30 min at 37°C. Cells were washed twice with 1× PBS, and fluorescent intensity of the cells was measured using in BD FACS verse in FACSuite software. PEG-Catalase (Sigma, United States) was used to inhibit H_2_O_2_ mediated ROS generation.

### Minocycline Administration in the Animal Model

Adult BALB/c mice (P10 Pups) were used for all experimental procedures. Mice were randomly assigned to three groups: Control group (Mock); CHPV infected group (CHPV); CHPV and Minocycline treated group (CHPV + M). We used a previously described animal model of CHPV as described previously. BALB/c mice of either sex were injected with 5^∗^10^6^ pfu of CHPV virus through intraperitoneally. Mock animals received phosphate buffered saline (PBS). The twice daily dose of minocycline (Sigma, St Louis, MO, United States) of 22.5 mg/kg body weight was administered intraperitoneally (i.p.) after 8 hpi CHPV infection and continued for 3 days. Groups of five mice were killed 3 dpi time point either for tissue, protein or RNA. From third day onward animals started to show symptoms of CHPV including restriction of movements, limb paralysis, poor pain response, whole body tremor, piloerection, and hindlimb paralysis. After 3 dpi all animals succumb to death. All experiments were performed according to the protocol approved by the Institutional Animal Ethics Committee of National Brain Research Center (NBRC).

### RNA Isolation and Real-Time PCR (qPCR)

Mouse brain tissue and harvested HT22 cells were homogenized using trizol reagent as per manufacturers’ protocol (TRI reagent, Sigma, United States). For qPCR analyses, cDNA was synthesized using Advantage RT-for-PCR kit (Clontech Laboratories, CA). Oligonucleotide primers specific for N-Protein was used: forward 5′-ACC TGG CTC CAA ATC CAA TAC-3′ and reverse 5′-GGT GGA TCA GAC GGA GAG ATA-3′. Power SYBR Green PCR master mix (Applied Biosystems) was used for the experiment. The qPCR results were analyzed as per the user manual guidelines.

### Immunofluorescence

CHPV protein staining and LC3B (Abcam, United States) was performed on different brain sections. Anti-Rabbit Alexa fluor 488 (1:1000; Molecular Probes, Invitrogen, United States) was probed with the secondary antibody of CHPV protein. The corresponding secondary antibodies Fluorescein Isothiocyanate (FITC, 1:200; Vector Laboratories, United States) for N-protein were used.

### JC-1 Staining

The lipophilic cation JC-1 was used to assess the mitochondrial status following CHPV infection. According to the manufacturer (Thermo Fisher Scientific, United States), JC-1 changes its fluorescence reversibly from green (monomeric status) to orange (multimeric status) when the mitochondrial membrane potential is high. Samples were diluted with Tris buffer down to 30 × 10^6^ cells/ml, and 500 μl were transferred to a polypropylene tube. 0.5 μl of JC-1 stock solution (3 mM JC-1 in DMSO) was added. The tubes were kept in a water bath at 38°C for 40 min. Staurosporine (Sigma, United States) at 2.5 μM was added as a positive control for the experiment.

Samples were analyzed on a BD Facs Verse flow cytometer (Becton Dickinson Immunochemistry Systems; San Jose, CA, United States).

### FasL Staining

HT22 cells were plated at the density of 5 × 10^5^ cells per plate. Cells were Mock-treated, CHPV treated and CHPV + Minocycline treated and then harvest at 8 hpi. Cells were then properly washed with 1X wash buffer (BD Pharminagen, United States) at 300 g for 5 min. After washing cells were incubated with 5 ul of FasL-FITC conjugated (BD Pharminagen, United States) antibody for 15 min. Cells were then washed and was analyzed in BD FACS Calibur.

### Annexin V and Propidium Iodide Staining

Cells were harvested with ice-cold 1X PBS from various experiments to analyze cellular apoptosis and resuspended in 1X binding buffer solution provided with fluorescein isothiocyanate (FITC)-annexin V apoptosis detection kit I (BD Pharminagen, United States). By following the manufacturer’s instruction, we stained cells with FITC annexin V and propidium iodide (PI). Data were acquired and analyzed using BD FACSuit Software in FACS Verse (Becton, Dickinson, San Diego, CA, United States).

### Live Cell Imaging

Calcium concentration in the cell was estimated using live cell imaging. Cells were incubated with Fluo 4 AM (4 μM) dye for 30 min and then were washed twice with HEPES buffer for 5 min each, followed by 30 min of incubation in calcium containing HEPES buffer. Cells were then washed twice and infected in calcium free HEPES buffer media for calcium level measurement. Fluorescence intensity was then measured using Zeiss Axio observer.z1 microscope Yokogawa CSU-XA1 unit in ZEN Blue software for 15 min after virus addition. In all assays, including chelators, cells were treated 2 h before CHPV infection and drug concentrations were maintained throughout infection

Mitosox (Thermo Fisher Scientific, United States) was performed according to manufacturer’s protocol. In short, Cells were washed twice with 1X PBS and then incubated with dye for 15 min at 37°C followed by flouroscence measurement at Absorption/emission maxima: ∼510/580 nm.

### Calcium Measurements by Flow Cytometry

Changes in the intracellular cytosolic Ca^2+^ concentration were measured with the calcium-sensitive dye Fluo-4AM (Molecular Probes) in BD FACS verse. Briefly, HT-22 cells were seeded in the density of 1X10^5^ in 12 well plate. The next day, the confluent monolayers were changed to serum-free media and loaded with 4uM Fluo-4AM dye. The cells were then incubated with either culture medium, CHPV at an MOI of 0.1. Then after desired time point, cells were washed once in 1X PBS and reading was measured in BD FACS Verse in FACSuit. To check the effect of virus within minutes of infection Fluo-4AM loaded cells were collected and measured for response and then again virus was added to the tube and was measured in slow flow rate to get the shift after virus addition.

### Docking Study

The three-dimensional models of Angiotensin II interacting with CHPV protein were generated using the protein–protein docking program (ClusPro)^1^. The interaction models were evaluated using lowest energy values (Comeau et al., 2004). PDB codes for G-protein of CHPV and angiotensin were downloaded from the PDB website.^2^

### Immunoprecipitation Assay

Treated and untreated HT22 neuronal cells were lysed with cell lysis buffer (50 mM Tris buffer, pH 7.4, containing 150 mM NaCl, 5 mM EDTA, 1% NP-40) with freshly added protease inhibitors (1 mg/ml aprotinin, 1 mg/ml leupeptin, 1 mg/ml pepstatin, and 1 mM PMSF) and phosphatase inhibitors (20 mM NaF and 1 mM orthovanadate). Lysates were co-immunoprecipitated with 5 μg of anti-CHPV antibody for overnight at 4°C and incubated with protein A Sepharose beads (Sigma) for 2 h at 4°C. The immunocomplexes were then washed and probed by western blotting using anti-angiotensin II (Rockland, United States) antibody as well as anti-CHPV antibody.

### Statistical Analysis

Experiments with paired treatment were analyzed by *t*-tests. Experiments with >2 treatments were analyzed by ordinary one-way analysis of variance (ANOVA) as appropriate with Holm-sidak correction for multiple tests. Prior to analysis data were tested for adherence to normality using the Shapiro-Wilk normality test. All analyses were conducted using GraphPad 13.0.

## Results

### Minocycline Delayed Replication of Chandipura Virus

**FIGURE 1:**
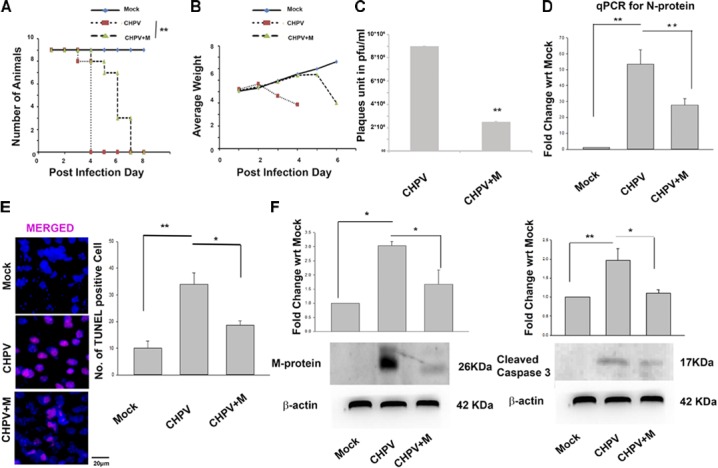
Minocycline treatment reduced the severity of infection and increases survivability in Balb/c mouse. **(A)** Kaplan-Meier plot showed an increase in survivability of animals after minocycline (CHPV+M) treatment by 48–72 h (2–3 days). **(B)** Growth plot showed a comparatively delayed decrease in weight of minocycline treated group against only CHPV infected group. **(C)** Plaque assay was performed on Vero cells inoculated with brain homogenate obtained from CHPV infected and CHPV infected-minocycline treated groups. Significant low number of plaques was observed in Vero cell culture infected with minocycline treated group when compared to the untreated group. **(D)** qPCR analysis showed a significant reduction in CHPV N-protein mRNA level after minocycline treatment when compared to only CHPV infected group. **(E)** TUNEL assay showed a decrease in TUNEL positive cells after minocycline treatment in CHPV infected, and minocycline treated brain sections. Bar graph was plotted denoting number of TUNEL positive cells in different samples. **(F)** Western Blot for Viral M-protein and cleaved Caspase 3 showed significant decrease in expression after minocycline treatment in CHPV infected brain samples, which was quantified by densitometry graph. Validation of results were done by 3 independent experiments with at least 4 animals in each group. ^∗^*p* < 0.05, ^∗∗^*p* < 0.01.

Minocycline treated (CHPV+M) animals succumbed to CHPV infection at 6–7 days post-infection (dpi) compared to untreated animals that die at 3–4 dpi (**Figure 1A**). Drastic loss of weight is one of the symptoms of CHPV infection as described previously (Ghosh et al., 2013). It was observed that compared to the CHPV group onset of weight loss in minocycline post-treated animals was delayed by approximately 48 h (**Figure 1B**). These two observations intrigued us to know the reason behind the enhanced survivability of the minocycline treated group post-CHPV infection. A significant reduction in plaques was observed in minocycline treated groups when brain homogenate from both the groups were used to infect Vero cells in culture (**Figure 1C** and Supplementary Figure S1C). Reduction in CHPV N-protein (CHPV Nucleation Protein: for replication of viral RNA) replication was observed in minocycline treated groups from real-time PCR at 3 dpi (3 dpi time post- CHPV infection denotes appearance of neurological symptoms; **Figure 1D**). Neurodegeneration has been previously linked to replication of CHPV (Ghosh et al., 2013). Significant reduction in TUNEL positive cells in minocycline treated group was observed as compared to CHPV infected group (**Figure 1E** and Supplementary Figure S1A). Western blots of M-protein of CHPV and cleaved Caspase 3 showed the significant reduction in expression in minocycline treated sample (**Figure 1F** and Supplementary Figure S3B). Concomitantly these observations helped us to conclude that minocycline hinders the replication of the virus in neurons and hence neurodegeneration *in vivo*.

### Minocycline Decreased Viral Load in HT22 Cells

**FIGURE 2:**
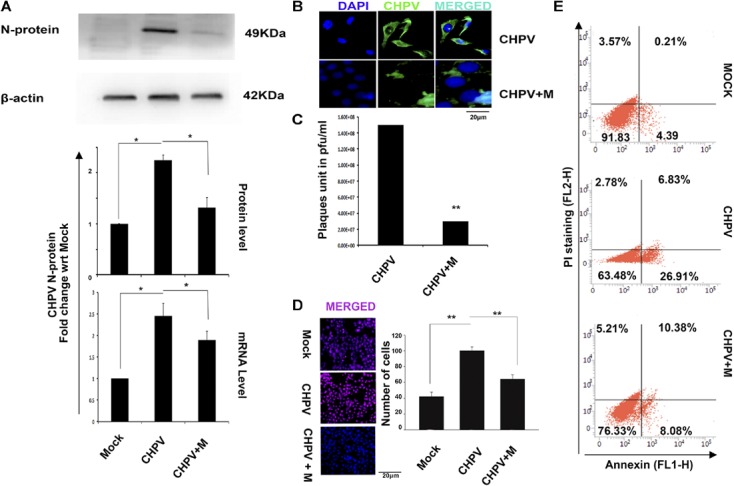
Minocycline treatment reduced CHPV replication and apoptosis in HT22 cells infected with CHPV virus. **(A)** CHPV N-protein expression decreased in minocycline (CHPV + M) treated group when it was compared to CHPV infected sample. qPCR analysis concomitantly supports the Western Blot. **(B)** Immunocytochemistry for N-protein showed comparatively less N-protein positive cells in minocycline treated group as compared to CHPV infected cells. **(C)** Plaque assay showed a reduced number of plaques in minocycline treated group. **(D)** TUNEL staining showed that minocycline treatment rescued cell death. **(E)** Annexin-PI staining showed a reduction in Annexin-V positive cells in minocycline treated group when compared to CHPV infected group. All experiments were repeated at least three times before concluding. ^∗^*p* < 0.05, ^∗∗^*p* < 0.01.

Since CHPV has been proven to selectively infect neurons in the brain, to prove our hypothesis, we used HT22 cells that are neuronal hippocampal cell lines. HT22 cells were infected and treated according to protocol and then processed for experiments at 12 h post infection (hpi) (12 hpi time post-CHPV infection denotes cellular apoptosis). Cytotoxicity of minocycline was performed using MTT assay (Supplementary Figure S3A) Western Blot analysis of the CHPV N-protein showed a significant downregulation in expression as compared to CHPV infected group (**Figure 2A**). Densitometry graph showed an increase in viral N-protein by more than 2-fold with respect to (w.r.t) mock that decreased significantly in minocycline treated group. The data was further confirmed at RNA level by real-time PCR (**Figure 2A**). N-protein staining in CHPV infected group showed very low signal in minocycline treated group (**Figure 2B**) suggesting a decreased viral load. Plaque assay was performed by re-infecting Vero cells by supernatants obtained from the groups: CHPV infected, and CHPV infected post-treatment with minocycline; further confirmed the efficacy of minocycline drug against CHPV replication *in vitro* (**Figure 2C**).

Similar to our *in vivo* results TUNEL staining showed significant decrease in TUNEL positive cells in minocycline treated group when compared to CHPV infected group (**Figure 2D** and Supplementary Figure S1B). The cell death *in vitro* was further tested morphologically (Supplementary Figure S1B) and through Annexin- PI staining (**Figure 2E**). These data cumulatively confirmed that minocycline reduced viral load and apoptosis in HT22 cells.

### Minocycline Inhibited Apoptosis Through the Extrinsic Apoptotic Pathway

**FIGURE 3:**
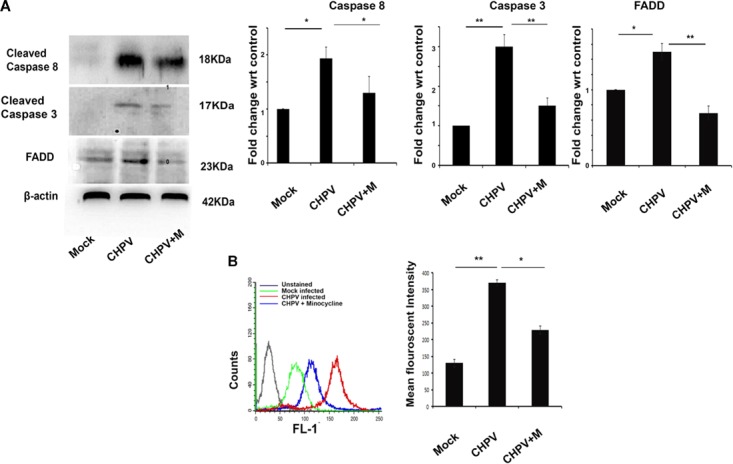
Effect of minocycline on the protein expression of extrinsic apoptotic signaling molecules. **(A)** Expression of FADD, Casp8 and cleaved Casp3 was found to be upregulated in CHPV infected group, while it decreased in minocycline (CHPV + M) treated group *in vivo*. **(B)** FasL level in HT22 cells was analyzed using flow cytometry to check its expression level. A significant decrease in minocycline treated group was observed when compared against only CHPV treated cells. Graph showed mean fluorescent intensity of FasL expression. FasL level was monitored by FL-1 channel. Three independent experiments were performed to reach a conclusion. ^∗^*p* < 0.05, ^∗∗^*p* < 0.01.

The decrease in FADD and cleaved caspase 8 in mouse brain (3 dpi) tissue post minocycline treatment as compared to only CHPV infected samples indicated that minocycline treatment was inhibiting apoptosis through the extrinsic pathway (**Figure 3A**). Western blot analysis showed a decrease in cleaved Caspase 3 (Casp3) level in minocycline treated sample as compared to the only CHPV infected group (**Figure 3A**). A significant decrease in FasL level in minocycline treated group as compared to only CHPV infected group in HT-22 samples at 12 hpi (**Figure 3B**). Mean fluorescence intensity (MFI) graph was plotted for each sample showing the significant difference in fluorescent intensity among samples.

### CHPV Induced Mitochondrial Dysfunction

**FIGURE 4:**
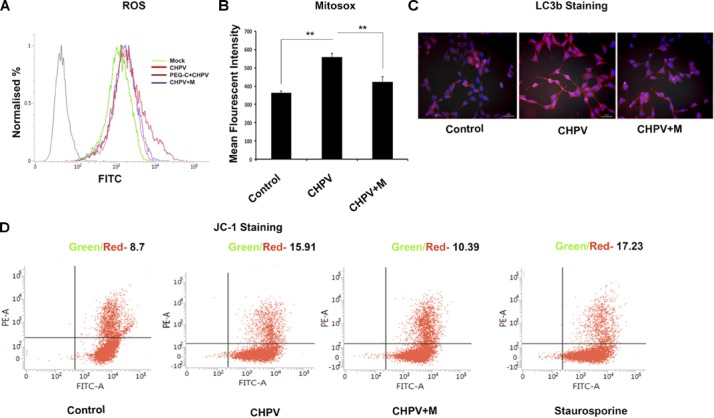
CHPV infection induced mitochondrial dysfunction in the neuronal cell line. **(A)** ROS was measured at 4 hpi and was found to be increased against the mock infected group. Minocycline (CHPV + M) treated group showed decreased level of ROS generation against CHPV infected group. **(B)** Superoxide production level was measured using Mitosox. MFI was maximum in CHPV infected sample and was decreased in minocycline treated group. **(C)** LC3B staining, a marker for autophagy showed more expression in CHPV infected group as compared to minocycline treated samples, suggesting minocycline treatment inhibited induction of autophagy. **(D)** JC-1 staining showed more green:red signal in CHPV infected group indicating altered mitochondria. Compared to the CHPV infected group, minocycline treated group showed a reduced ratio of green: red signal signifying its treatment rescued mitochondrial alteration. Three independent experiments were performed to reach a conclusion. ^∗^*p* < 0.05, ^∗∗^*p* < 0.01.

Minocycline has been previously reported to act as an antioxidant by increasing activity of superoxide dismutase. Experiments in our lab showed a heightened release of ROS in CHPV induced neuronal apoptosis (Supplementary Figure S1D), and inhibition of ROS inhibited viral replication (Ghosh et al., 2016), blocking the ROS activity using NAC inhibited apoptosis. Mitochondrial dysfunction is one of the critical factors in infection-induced apoptosis. Mitochondrial ROS generation is considered to be an essential phenomenon in inducing apoptosis was checked. ROS generation in HT22 cells post 3 hpi was measured using 2′,7′-dichlorofluorescin diacetate (DCFDA) that showed more fluorescent intensity in CHPV infected sample that decreased sharply in minocycline treated group (**Figure 4A** and Supplementary Figure S2C). MFI showed significant decrease in ROS level in minocycline treated sample (Supplementary Figure S2A). Further, we checked whether the ROS generation was H_2_O_2_ mediated. Cells were treated with PEG-catalase 2 h before infection that showed a decrease in ROS secretion, suggesting H_2_O_2_ is a significant player in this mode of ROS generation (**Figure 4A** and Supplementary Figure S2A). Mitosox was used to check the level of superoxide generation in live cells. Fluorescence measurement showed enhanced level as compared to mock that decreased significantly in minocycline treated group (**Figure 4B**). During mitochondrial dysfunction, autophagy gets induced. The microscopy figures at 20X showed a decrease in LC3B staining in minocycline treated sample when compared to CHPV treated samples (**Figure 4C**). JC-1 (5, 5′, 6, 6′-tetrachloro-1, 1′, 3, 3′-tetraethylbenzimidazol-carbocyanine iodide) is a lipophilic fluorescent dye that distinguishes polarized from de-polarized mitochondria that infer the health of the cells. JC-1 in monomeric form emits green fluorescence when excited with a light of wavelength ∼488 nm, but in a healthy cell, it can get incorporated into the mitochondrial membrane, where it forms J-aggregates due to the physiological membrane potential of mitochondria and its fluorescence shift from green to red. Due to oxidative stress, the mitochondrial potential reduces, and the J-aggregates disassociate to release JC-1 in monomeric forms into the cytoplasm shifting fluorescence from red to green again. JC-1 staining was used to determine the mitochondrial potential through the course of CHPV infection in neurons. Flow cytometry staining result showed that post 6 hpi (6hpi time post-CHPV infection denotes viral replication phase within cells) mitochondria showed more of green staining suggesting mitochondrial death in CHPV infected (**Figure 4D**).

### Intracellular Calcium Played a Significant Role in CHPV Infection

**FIGURE 5:**
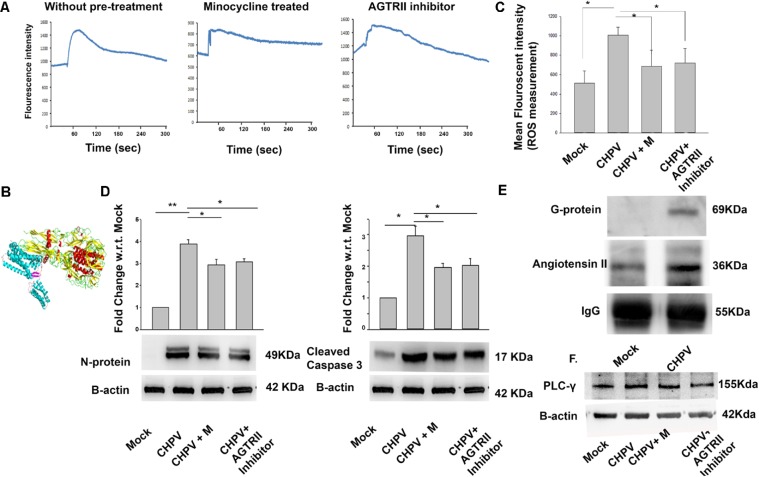
CHPV induced calcium influx into cell leading to ROS generation. **(A)** 60 s of baseline were recorded, and then virus was added to observe the effect of CHPV in HT22 cells. Baseline varies from sample to sample so only change from baseline to active state was calculated. Fluo-4 AM dye showed increase in intracellular calcium that lasted for 5 min. Then the same recording was done in the presence of candesartan, also known as angiotensin II receptor antagonist (AGTRII inhibitor). Calcium level in respect to base line increase was not significant. Calcium signaling in minocycline(CHPV + M) treated group was checked and was found to be similar to AGTRII inhibitor treated group suggesting minocycline acts on intracellular calcium level. **(B)** *In silico* study showed an interaction between angiotensin II and CHPV G-protein. **(C)** ROS level decreased in groups treated with AGTRII inhibitor or minocycline. **(D)** After AGTRII inhibitor treatment cleaved Caspase 3 level and viral load also decreased. **(E)** Western blot for proteins pulled by CHPV G-protein showed increased expression of Angiotensin II in CHPV treated group as compared to Mock-infected.**(F)** Western blot image shows phosphorylation of PLC-γ suggesting the involvement of PLC-γ in calcium signaling. Three independent experiments were performed to reach a conclusion. ^∗^*p* < 0.05, ^∗∗^*p* < 0.01.

A significant rise in intracellular calcium (Ca^2+^) secretion was observed after CHPV infection that lasted for 5-10 min (**Figure 5A**). The difference between thresholds to peak was observed. As a part of control experiment, we used specific Ca^2+^ chelator i.e., EGTA (ethylene glycol-bis(beta-aminoethyl ether) an extracellular Ca^2+^ chelator as well as BAPTA-AM (15 μM) (1,2-bis(o-aminophenoxy)ethane-N,N,N′,N′-tetraacetic acid) an intracellular Ca^2+^ chelator to determine whether the Ca^2+^ release was extracellular or intracellular. A significant decrease in BAPTA-AM treated sample suggested intracellular Ca^2+^ contributed to increasing cellular Ca^2+^ level (Supplementary Figures S2B,D). GPCRs (G-protein coupled receptors) are involved in efflux of stored Ca^2+^ to cytoplasm (Cordeaux and Hill, 2002; Billups et al., 2006). *In silico* studies were performed to predict the interaction of viral protein (G-protein) and GPCRs. Angiotensin II receptor was identified to be the best match (**Figure 5B**). Angiotensin was selected by the enthalpy of interaction between two proteins. Then, we checked for cellular calcium in the presence of angiotensin II antagonist Candesartan cilexetil. Our data showed a decrease in calcium level from basal level to peak after addition of virus (**Figure 5A**). A significant decrease of ROS in Angiotensin II receptor antagonist as well as in Minocycline treated sample was observed (**Figure 5C**). This data suggested us that Angiotensin II receptor plays a role in calcium secretion and its antagonist decreased calcium levels. Hence Angiotensin II level and minocycline may be working in a similar fashion. To check if Candesartan Cilexetil (AGTRII inhibitor) has any effect on viral replication and apoptosis, we performed western blot for CHPV N-protein and Casp 3 (**Figure 5D**). Both the blots showed a significant reduction in expression of both these proteins in samples treated with AGTRII inhibitor.

Co-immunoprecipitation study determined the possible interaction at the molecular level with the CHPV Glycoprotein G-protein. Increase in expression of Angiotensin II was evident from our data (**Figure 5E**). Densitometry of blots are presented in Supplementary Figure S3C. Significant amount of Angiotensin II was found to be coupled with G-protein in the infected samples compared to mock when normalized to IgG as a loading control. Hence, we wanted to analyze signaling involved in Ca^2+^ increase via Angiotensin II receptor. PLC-γ (Phospholipase C) level was analyzed as it is a known protein to be involved in the pathway. Phosphorylation of PLC-γ is involved in Angiotensin II mediated Ca^2+^ increase (**Figure 5E** and Supplementary Figure S3D).

### CHPV Infection Leads to Phosphorylation of p38

**FIGURE 6:**
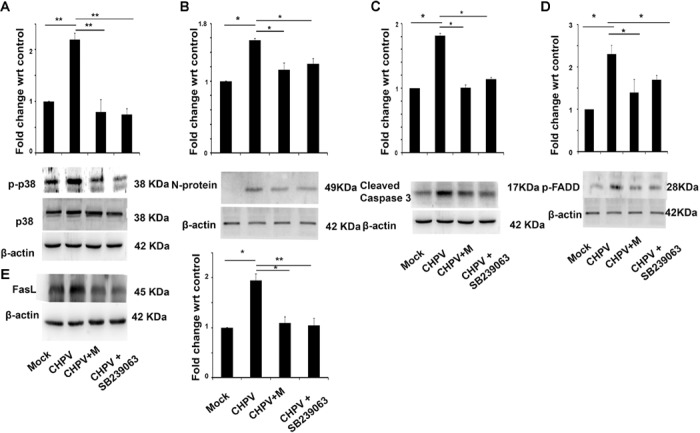
Phosphorylation of p38 is an important event in CHPV infection. **(A)** After CHPV infection phosphorylated p38 level increased suggesting a role of p38 in CHPV infection. In minocycline (CHPV + M) treated group p-p38 level decreased significantly with respect to CHPV infected samples. **(B)** Viral protein level was checked after inhibiting p38 by known inhibitor SB239063 and was found that viral protein level decreased in minocycline treated samples as well as in p38 inhibitor-treated samples. **(C)** Cleaved Caspase 3 level was checked to understand the role of p38 on apoptosis in CHPV infection. It was found to decrease in minocycline treated sample as well as p38 inhibitor sample. **(D)** FADD expression level was checked after p38 inhibitor as its involvement is well established in CHPV induced apoptosis. Phosphorylated FADD level decreases significantly in Minocycline as well as SB239063 treated group. **(E)** FasL level was checked in the presence of p38 inhibitor. Western blot result showed a decrease in FasL level in minocycline, as well as SB239063, treated samples. Three independent experiments were performed to reach a conclusion. ^∗^*p* < 0.05, ^∗∗^*p* < 0.01.

Mitogen-activated protein kinases are linked to apoptosis induction and are reported to get activated in several RNA virus infection (Wei et al., 2009; Ceballos-Olvera et al., 2010). We found that post-CHPV infection in HT22 cells, p38 got phosphorylated. Minocycline treatment to cells post infection decreased phosphorylation of p38 (**Figure 6A**). It was interesting to see that minocycline, as well as the p38 inhibitor (SB239063), were efficient in inhibiting viral load as well as Caspase 3 inhibition, suggesting the role of p38 in CHPV infection and apoptosis (**Figures 6B,C**). As p38 induces apoptosis through FasL mediated pathway and CHPV shared the same, FADD and FasL level in the presence of virus, minocycline and SB239063 were examined. Western blot analysis showed a significant decrease after inhibition of p38 phosphorylation (**Figures 6D,E**).

## Discussion

Our observations on minocycline treatment to CHPV infected Balb/c mice showed delayed onset of symptoms and death. Experimental data although showed minocycline potentially reduced viral load and neuronal apoptosis in mouse brain but was not competent enough to evade death in the infected animals (**Figure 1**). To understand the antiviral mechanistic pathway of minocycline, we experimented on HT22 cells, and the data was consistent with the *in vivo* results (**Figure 2**). FasL that is a well-known inducer of apoptosis, was observed to down-regulate in fluorescence intensity after minocycline treatment. Similarly, other markers of extrinsic apoptotic pathways were also found to decrease in the minocycline treated group (**Figure 3**).

Our study also revealed that ROS generation increased significantly after CHPV infection. In relation to that mitochondrial health was assessed with the help of mitosox and JC-1 suggesting CHPV induced mitochondrial dysfunction in HT22 cells. After minocycline treatment, ROS generation was relatively less and so as mitochondrial dysfunction (**Figure 4**). Our data suggested if ROS can be blocked during CHPV infection then virulence of CHPV can get mitigated. Although there are ample reports that indicate that excessive ROS generation can be disastrous to cells and are associated with apoptosis, specific mechanism involved in ROS-mediated apoptosis has not been identified. Calcium release or increase in calcium within cells is a causative agent of oxidative stress and hence release of ROS.

In our present work, we have shown that viral infection leads to enhanced cellular calcium. Our *in silico* data of protein–protein interaction suggested the best result with G-protein coupled receptor specifically with Angiotensin II. Angiotensin II receptor antagonist Candesartan cilexetil treated cells showed minimum change in cellular calcium as compared to only CHPV group. A similar observation was seen in case of minocycline treated group after virus infection suggested that minocycline has some role to play in calcium signaling. We also witnessed an increase in calcium with time in virus infected samples that decreased in minocycline treated samples (Supplementary Figure S2E). This might be one explanation of mechanism of action of minocycline in our case but to understand this phenomena an in-depth study is required. Our experiments revealed a direct molecular interaction between Angiotensin II and CHPV G protein that showed an increase in intracellular calcium levels in the cell (**Figure 5E**). Enhancement of cytoplasmic calcium may be attributed to synchronized or independent activation of PKC-δ or PLC-γ. Two key pathways established related to Angiotensin II induce calcium secretion are through PKC-δ and PLC-γ. To understand the pathway, we measured the expression of both the protein in CHPV infected samples and found only increase in PLC-γ whereas no change was found in PKC-δ (Data not shown).

Role of the inflammatory molecule is well established in CHPV induced neurodegeneration (Verma et al., 2016). JNK and p38 are stress-activated MAP kinases that are preferentially activated by cell stress-inducing signals, including environmental stress, oxidative stress, and toxic chemical insults (Kyriakis et al., 1994; Johnson and Lapadat, 2002; Weston and Davis, 2007). Sustained activation of JNK or p38 is entailed in the induction of many forms of neuronal apoptosis in response to a variety of cellular damage (Namgung and Xia, 2000). p38 MAPK is downstream of CAMKII in a signaling pathway by which an increase in cytosolic Ca^2+^ leads to increase in PGC-1α expression and mitochondrial biogenesis in skeletal muscle (Rose et al., 2004).

**FIGURE 7:**
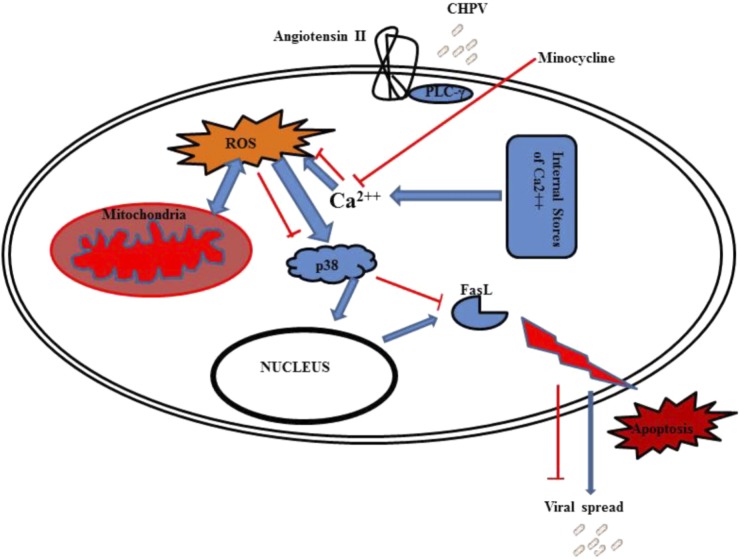
Graphical representation shows CHPV interacts with angiotensin II that leads to phosphorylation of PLC-γ. This PLC-γ induces calcium stored in organelles to come out in cytoplasm that leads to ROS generation. ROS induces mitochondrial dysfunction which further elevate ROS. This leads to activation of p38 protein which helps in FasL production through some unknown mechanism. FasL then induces apoptosis through extrinsic apoptotic pathway.

Now the final question was how does calcium increase modulates neuronal apoptosis? CHPV induce Fas-mediated neuronal apoptosis, and our data showed that minocycline inhibited CHPV induced neurodegeneration through the Fas-mediated pathway. p38 is involved in CHPV induced FasL production that lead to initiation of neuronal death post-CHPV infection. In this work, we have shown that inhibiting p38 phosphorylation using specific inhibitor inhibits apoptosis and also viral load. Similarly, minocycline that has been previously reported to have anti-apoptosis role was found to restrict p38 phosphorylation and hence FasL production (**Figure 7**). Although the pathway by which p38 induced the expression of FasL is not described in present work, but reports suggest that p38 mediate FasL expression, including AP-1, NF-κB, and NF-AT (Kasibhatla et al., 1998; Faris et al., 1998).

To summarize the findings of this report, CHPV G-protein binds to Angiotensin II receptor that further activates PLC-γ that leads to increase in intracellular Ca^2+^. This enhanced intracellular Ca^2+^ levels induces the release of ROS and other free radicals from the mitochondria. Enhanced rise in ROS levels results in mitochondrial dysfunction. ROS also activates p38 molecules by phosphorylating them that further enters the nucleus to trigger the production of FasL and hence apoptosis through the extrinsic apoptotic pathway (**Figure 7**). Minocycline was able to partially affect ROS generation by acting as a Ca^2+^ chelator. Although our report does not comprehensively define as to how minocycline chelates calcium but these events affected the propagation of CHPV by inhibiting apoptosis of cells. Minocycline has been reported to combat viral replication and stimulate anti-apoptotic pathways in both *in vivo* and *in vitro* models in this report, but the exact reason as to why it failed to bring about complete survival in the animal model is not known and would require further investigations.

## Author Contributions

AV carried out acquisition and analysis of the data and drafted the manuscript. SG conceived and helped in drafting the manuscript. AB conceived and designed the study and helped in drafting the manuscript. All authors have read and approved the final manuscript.

## Conflict of Interest Statement

The authors declare that the research was conducted in the absence of any commercial or financial relationships that could be construed as a potential conflict of interest.

## Footnotes

1 https://cluspro.bu.edu

2 http://www.pdb.org
