# Supplementary material for: Chandipura Virus Induced Neuronal Apoptosis via Calcium Signaling Mediated Oxidative Stress
Source: Front Microbiol. 2018 Jul 6;9:1489. doi: 10.3389/fmicb.2018.01489 (PMC6043780; doi:10.3389/fmicb.2018.01489)
Supplement: Supplementary file 1 [file Data_Sheet_1.pdf]

Chandipura virus Induced Neuronal Apoptosis via Calcium Signalling mediated oxidative stress

Abhishek Kumar Verma, Sourish Ghosh#, Anirban Basu@

National Brain Research Centre, Manesar, Haryana-122052, India

Running Title: Calcium signalling in CHPV infection

@ To whom correspondence should be addressed:
Anirban Basu,
National Brain Research Centre,
Manesar, Haryana-122052, India.
Email: anirban@nbrc.ac.in

# Present Address – Cell Biology and Physiology Centre,
National Heart Lung and Blood Institute, NIH, Bethesda, Maryland, USA

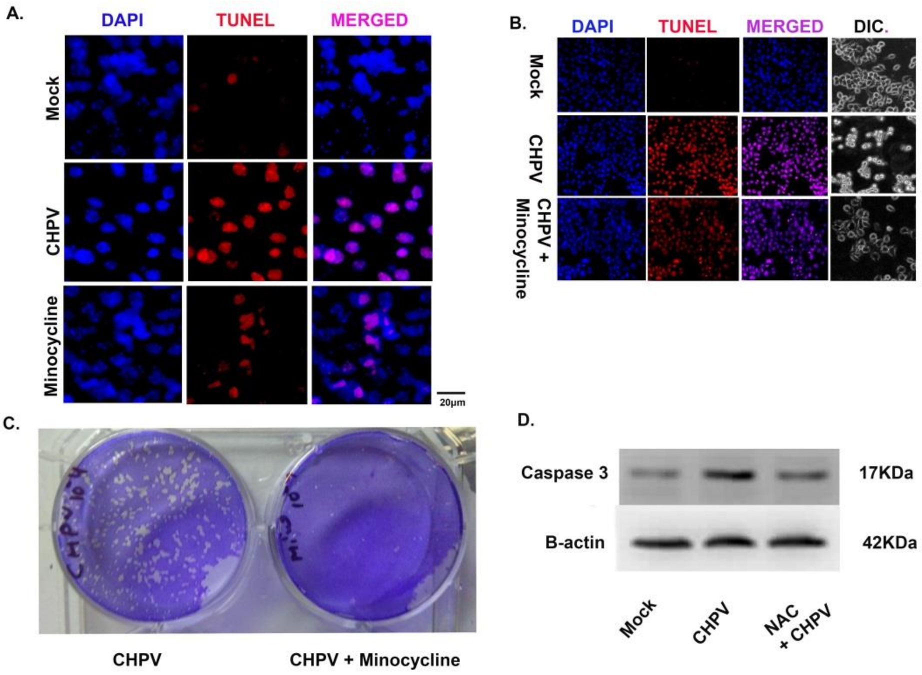

Supplementry Fig 1. A) Single panel image of TUNEL staining in brain section shows decrease in TUNEL positive cells in minocycline treated group as compared to CHPV treated group. Blue stain

shows DAPI whereas Red shows TUNEL stain. Pink shows merged image. B) TUNEL stain was further performed in HT22 cells. C) Plaque assay of viral load in was performed in presence and absence of minocycline shows minocycline reduces viral load. D) N-acetyl cysteine was used to inhibit ROS production and check its effect on neuronal apoptosis. Caspase 3 level was found to increase in CHPV infected sample whereas it decreases significantly in NAC treated CHPV infected samples.

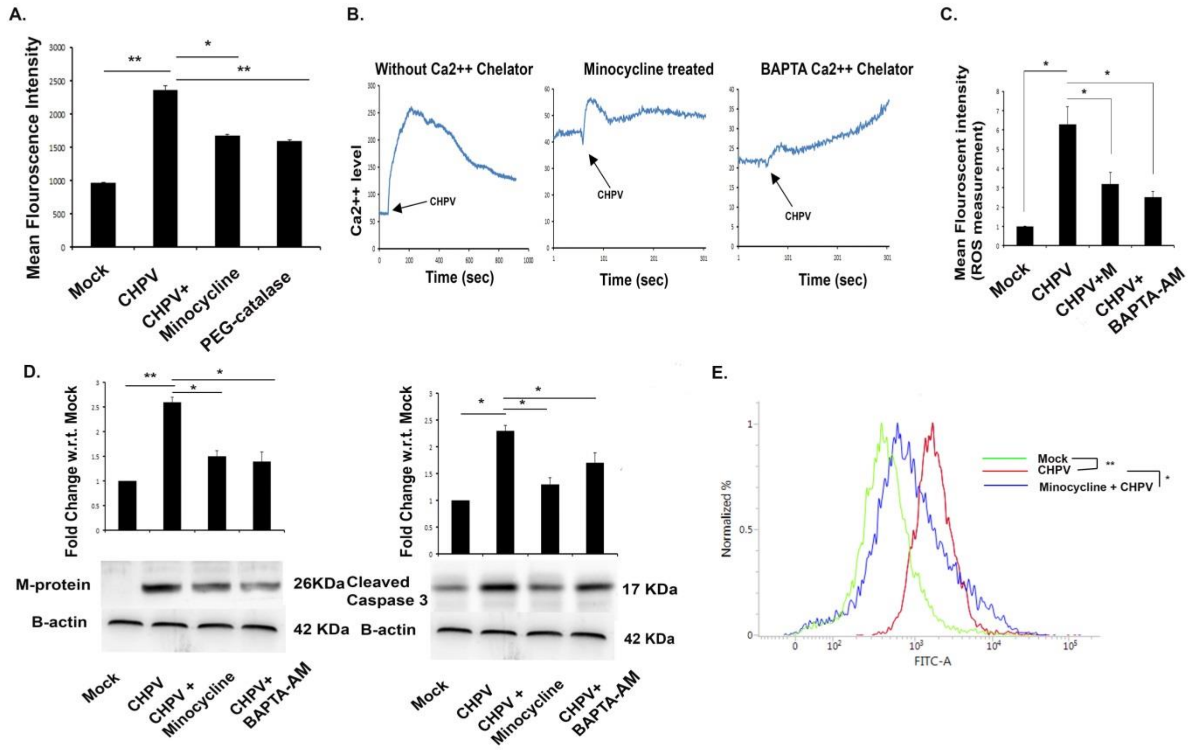

Supplementary 2. CHPV induces calcium influx into cell leading to ROS generation. A) Mean fluorescent Intensity graph was plotted for ROS measured using Flow cytometry. B) CHPV induces calcium influx into cell. 60 secs of baseline was recorded and then virus was added to observe the effect of CHPV in HT22 cells. Baseline varies from sample to sample so only change from baseline to active state was calculated. Fluo-4 AM dye showed increase influx of calcium in cytoplasm which lasted for 5 mins. Then the same recording was done in presence of BAPTA, a known calcium chelator and was found that after calcium chelation calcium level in respect to base line increase was not significant. Calcium signalling in minocycline treated group was checked and was found to be similar to BAPTA-AM treated group suggesting minocycline acts as intracellular calcium chelator. C) ROS level was checked to see if calcium has any effect on ROS generation. It was found that after blocking calcium using BAPTA-AM or minocycline, it decreases ROS level. D) After BAPTA-AM treatment cleaved caspase 3 level decreases and viral load also decrease. E) Calcium was measured using Fluo-4AM at 6hpi post infection. FACS analysis shows shift in CHPV infected sample as compared to mock infected which decreases in minocycline treated sample.

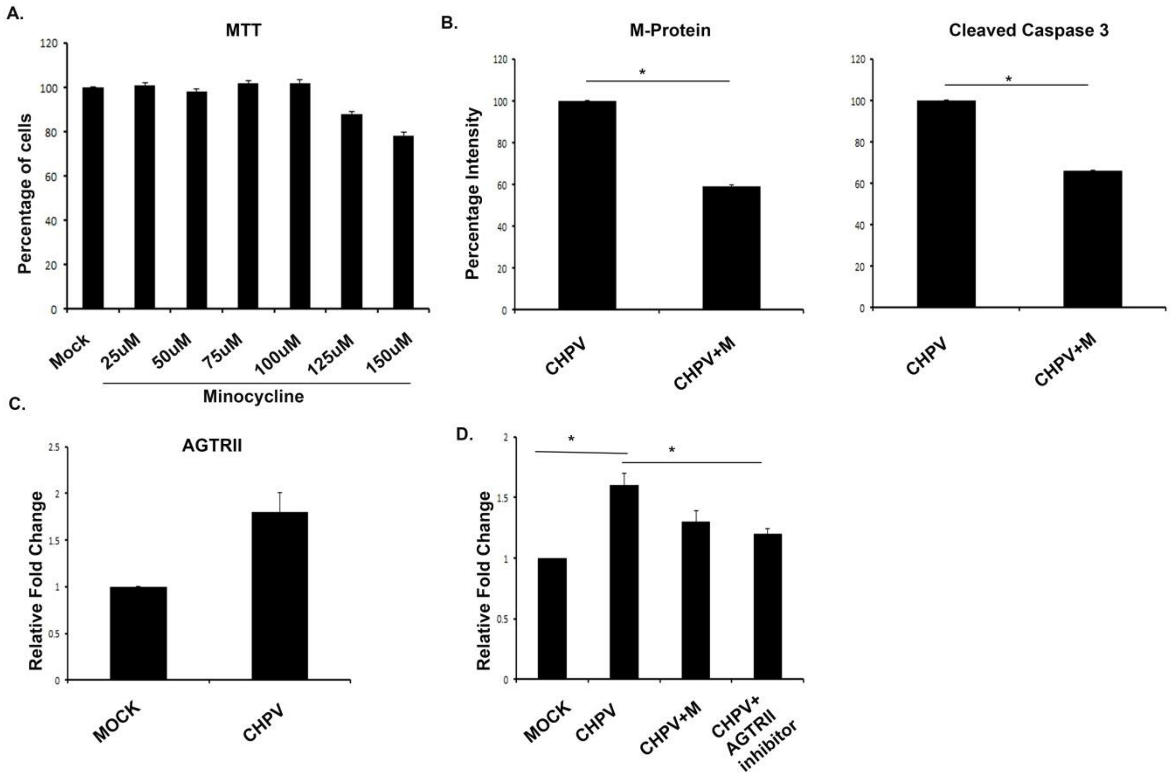

Supplementary 3. A). MTT assay was performed to check cytotoxicity of minocycline used for experiments. Our data shows minocycline doesn't have toxic effect at 75uM concentration used during cell treatment.

B). Effect of minocycline on cleaved caspase 3 and viral load was checked through immunoblot. Our data shows significant reduction in CHPV protein as well as cleaved caspase 3 in minocycline treated group as compared to CHPV trated groups.

C). Co-IP was performed for pull down of angiotensin II receptor (AGTRII). Densitometry data shows significant pull down of AGTRII in CHPV infected samples.

D). Immunoblot for PLC-γ shows increase in CHPV infected samples which decreases after AGTRII inhibitor suggesting role of Angiotensin II receptor in PLC- γ activation.
